# “Hard-To-Reach” or Hardly Reaching? Critical Reflections on Engaging Diverse Residents From Low Socio-Economic Status Neighborhoods in Public Health Research

**DOI:** 10.3389/ijph.2022.1605296

**Published:** 2023-01-05

**Authors:** Zeinab Aliyas, Patricia A. Collins, Shadé Chrun-Tremblay, Tevfik Bayram, Katherine L. Frohlich

**Affiliations:** ^1^ Institut de Recherche en Santé Publique, Université de Montréal, Montreal, QC, Canada; ^2^ Department of Geography and Planning, Faculty of Arts and Science, Queen’s University, Kingston, ON, Canada; ^3^ École de Santé Publique, Université de Montréal, Montreal, QC, Canada

**Keywords:** questionnaire survey, low socio-economic neighbourhoods, recruiting minorities, child studies, play street, school street

While socioeconomically disadvantaged populations are more likely to experience poor health, they are less likely to be represented in public health research [1–3]. This is particularly true in the case of low SES first-generation immigrant communities who may neither speak the language of their adopted country nor be computer literate. The low representation of such communities in research is in part due to the additional strategies needed to recruit and retain these populations, leading researchers to sometimes label socioeconomically disadvantaged immigrant populations as “hard-to-reach.” The labeling effectively blames these populations for their lack of engagement in research and can lead to further exclusion [2, 4]. Ensuring their inclusion in public health research should be prioritized in order to dismantle the structures that keep them in poor health in the first place [5]. In this regard, a number of studies have outlined the need for flexibility and tailoring of data collection methods but still, the methodological rigor of these studies is variable, and the body of evidence has significant gaps. Furthermore, approaches effective in one setting or one population may not be generalizable to another, which emphasizes the importance of more studies in varying communities.

This paper aims to explore the importance of using tailored methods in the recruitment of socioeconomically disadvantaged populations in order to increase the representation of these groups in public health research as well as some of the caveats of these methods. The recruitment we describe took place as part of a project entitled “Levelling the Playing Fields,” a population health intervention research project studying the effects of Play Street (PS) and School Street (SS) interventions on children’s free play, independent mobility, and active transportation. In this paper, we present the strategies we used to mitigate barriers to participation in low SES first-generation immigrant neighborhoods as well as the methodological implications we faced. As such, we argue that methodological tailoring requires continual readjustments throughout the process of conducting research in such communities in order to make participation more accessible. Our research suggests that “hard-to-reach” populations are not “hard-to-reach” *per se*, but in fact reachable if conventional public health research methods are adapted to the needs of their targeted populations.

From July to November 2021 we sought to recruit study participants from two socioeconomically disadvantaged neighborhoods in Montreal, Quebec, both of which contained important proportions of racialized people recently immigrated to Canada from the Global South. The target population was parent-child pairs from the neighborhoods where the SS and PS would be operating.

We had initially planned to collect the baseline data online, as this has become *de rigueur* for population studies. In the case of the prospective PS participants, we went door-to-door in the neighborhood with a community representative from a local organization to explain the intervention and recruit parents and children to participate in the online survey. In doing so, we discovered not only that people in this neighborhood did not have access to computers, but many of them did not know how to read or write in English and/or French, Canada’s two official languages. This was the wake-up call for us to change our strategy from administering surveys online to in-person data collection methods adapted to our target population. In our case, this entailed in-person recruitment with hard copy questionnaire completion assisted by an interpreter or community representative.

Similarly, for the recruitment of SS participants, our initial protocol was to have a school administrator randomly choose 60 student participants and request that their parents fill out the questionnaire online with their child. However, using this approach we were unable to reach 60 parent-child dyads and decided to distribute the remaining 24 surveys by having school administrators distribute them among the entire pool of students. We suspected that sending the hard copy versions *via* schools may make the parents feel like the request would be more legitimate and trustworthy, given that it came from a trusted school authority. Within 2 weeks, 60 families had filled out the questionnaire, twice the responses we had received using the earlier method, and took a tenth of the time required to obtain online questionnaires.

We conclude from our study that when working in low SES first-generation immigrant neighborhoods recruitment methods should not be chosen before going on-site, but instead, should be informed beforehand with the target population. Knowing the culture of the target population prior to data collection (even in the research design phase), thus avoiding the need for unplanned changes, project budgets can more accurately reflect the reality of data collection procedures, and participants will feel better understood and valued by researchers. We also found that recruitment through a mediator—whether a community organization or school administration—might be the most effective approach in low-SES, high-immigration neighborhoods. We found such mediation helps to build trust among the target community.

While our team was committed to adapting our methods to reach our sample size targets, the unplanned changes in our data collection strategies may have compromised the validity of the data collected (e.g., inaccurate translations, respondent fatigue, the influence of researcher’s presence and sample bias given the non-random nature of our sample), as well as the comparability of the data to other study sites where the survey was administered online. Researchers need to weigh their resources of time, financial budget, effort, and expertise to choose which of the recruitment interventions are truly ideal for the population they are attempting to recruit and ensuring the inclusion of these populations in public health research should be prioritized in order to dismantle the structures that keep them in poor health in the first place.
